# Multigenerational Consequences of Prenatal Exposure to Benzophenone-3 Demonstrate Sex- and Region-Dependent Neurotoxic and Pro-Apoptotic Effects in Mouse Brain

**DOI:** 10.3390/toxics12120906

**Published:** 2024-12-13

**Authors:** Karolina Przepiórska-Drońska, Andrzej Łach, Bernadeta Angelika Pietrzak-Wawrzyńska, Joanna Rzemieniec, Małgorzata Kajta, Agnieszka Wawrzczak-Bargieła, Wiktor Bilecki, Karolina Noworyta, Agnieszka Wnuk

**Affiliations:** 1 Laboratory of Neuropharmacology and Epigenetics, Department of Drug Addiction Pharmacology, Maj Institute of Pharmacology, Polish Academy of Sciences, Smętna 12, 31-343 Krakow, Poland; przepior@if-pan.krakow.pl (K.P.-D.); lach@if-pan.krakow.pl (A.Ł.); pietrzak@if-pan.krakow.pl (B.A.P.-W.); kajta@if-pan.krakow.pl (M.K.); 2 Department of Pharmaceutical Sciences, University of Milan, 20133 Milan, Italy; joanna.rzemieniec@unimi.it; 3 Department of Pharmacology and Brain Biostructure, Maj Institute of Pharmacology, Polish Academy of Sciences, Smętna 12, 31-343 Kraków, Poland; bargiela@if-pan.krakow.pl (A.W.-B.); bilecki@if-pan.krakow.pl (W.B.); 4 Affective Cognitive Neuroscience Laboratory, Department of Pharmacology, Maj Institute of Pharmacology, Polish Academy of Sciences, Smętna 12, 31-343 Kraków, Poland; noworyta@if-pan.krakow.pl

**Keywords:** apoptosis, benzophenone-3, environmentally pervasive chemicals, multigenerational changes, neurotoxicity, prenatal exposure

## Abstract

Benzophenone-3 (BP-3), commonly used as a UV filter in personal care products and as a stabilizer, is an alleged endocrine disruptor with potential neurodevelopmental impacts. Despite its abundance in the environment, the studies on its effect on brain development are scarce, especially in terms of multigenerational impact. In this work, for the first time, we examined neurotoxic and pro-apoptotic effects of BP-3 on mouse brain regions (cerebral cortex and hippocampus) in both the first (F_1_) and second (F_2_) generations after maternal exposure to environmentally relevant BP-3 levels. We found disregulated markers of cell damage (LDH, H_2_O_2_, caspase-3 and -8) and observed increased expression of pro-apoptotic *Fas*/FAS or *Fasl*/FASL. BP-3 exposure disrupted the BAX/BCL2 pathway, showing stronger effects in the F_1_ than in the F_2_ generation, with a dominance of extrinsic pathway (FAS, FASL, caspase-8) over intrinsic one (BAX, BCL2), suggesting that BP-3-induced apoptosis primarily operates via the extrinsic pathway and could impair brain homeostasis across generations. This study underscores the potential of BP-3 to increase multigenerational risks associated with disrupted neurodevelopment and highlights the importance of understanding its long-term neurotoxic effects.

## 1. Introduction

The knowledge of dangers associated with overexposure to sun radiation has become increasingly common [1,2,3,4,5,6], followed by increased demand and consumption of products protecting us from such rays. Photodegradation can also damage a wide variety of products, e.g., food packaging and building materials. Substances that answer this demand are chemical filters of UV, among which one of the most commonly used is benzophenone-3 (also known as BP-3, 2-hydroxy-4-methoxybenzophenone, oxybenzone, or 2OH-4MeO-BP). BP-3 is one of twelve members of benzophenones, a family of aromatic ketones, which are chemical absorbents of UV light. It is extensively used in the cosmetic industry, most notably as a component of sunscreen creams, as a scent stabilizer in perfumes, or as protection from photodegradation in a wide variety of products, e.g., plastic packaging, paints, and detergents [7,8,9]. Individual products differ in BP-3 concentrations; until 2017, the legally accepted concentration was 10% and in 2017, the ordinance of the EU reduced that threshold to 6% or 0.5% if the only use of BP-3 in a given product is to protect it from photodegradation [10]. The thresholds introduced in 2017 were maintained in the 2022 amendment to the EU Cosmetics Regulation [11]. In 2018 and 2019, Hawaii and Key West (Florida) banned the sale of sunscreens containing BP-3 due to its toxic and harmful impact on marine ecosystems [12]. This compound can be washed off into water from bodies covered in sunscreen products, contributing to environmental pollution. In particular, BP-3 was proven to exert negative effects on the reproduction of aquatic life and to evoke toxicity, contributing to the emergence of developmental abnormalities and enhanced mortality of marine fauna [8,13,14,15]. Importantly, BP-3 was also found to be accumulating in tissues of aquatic organisms, especially in adipose tissue, indicating that it can further accumulate through the food chain [16]. Such wide application of BP-3 translates to high levels of human exposure to this compound; solely in Europe, up to 1000 tons of BP-3 are being produced/imported annually [17].

According to the US Center for Disease Control and Prevention (CDC) and the EU Scientific Committee on Consumer Safety (SCCS), almost the entire world’s population is exposed to BP-3 [18,19,20,21,22]. Within 48 h of a single topical application of sunscreen containing BP-3, only 0.5% of the substance is removed from the body, while it still can be found in the urine after 96 h [23,24]. Recent reports indicate a link between urinary BP-3 levels and the prevalence of autoimmunity, as measured by antinuclear antibodies in adolescents and young adults [25]. Furthermore, BP-3 can enter the digestive system with potable water and other food products as a contamination originating from plastic packaging [26,27,28]. The respiratory system enables BP-3 entry with inhaled dust [29]. Additionally, BP-3 is lipophilic, which allows for its accumulation in adipose tissue [8]. This was demonstrated in a populational study on New York habitants, where a maximum concentration of up to 5 mg of BP-3 per kilogram of fat tissue was observed [30]. It was shown that BP-3 can cross the blood–brain barrier (BBB), since its presence was detected in *post mortem* adult brains [31]. This observation was confirmed by previous research, including our team using a new in vitro model of the BBB that consists of the primary culture of Wistar rat brain capillary endothelial cells, pericytes, and astrocytes [32,33,34]. It is also important to note the threats that BP-3 poses for prenatally developing organisms, since it easily crosses the placenta and its presence has been confirmed in amniotic fluid, placental tissue, the umbilical cord, and fetal blood [35,36,37]. Exposure to a high concentration of BP-3 follows a human into the postnatal period as it has been detected in human breast milk [8,35,36,37,38,39,40]. In addition, it is believed that BP-3 reaches significantly higher concentrations in newborns and young organisms, due to the low activity of their metabolic enzymes in comparison to adolescent and adult organisms [41,42]. Finally, human studies on prenatal exposure to BP-3 showed the association between maternal serum concentrations of BP-3 in pregnancy and reduced Leydig cell function in adult sons, also indicating an impact of BP-3 on the reproductive system [43].

To understand the meaning of studying the influence of a given substance on the cells, it is crucial to firstly become acquainted with its terminal effects, which can be potential outcomes of exposure to said substance. Apoptosis is a form of programmed cell death resulting in cell elimination that is mediated via DNA fragmentation and apoptotic body formation. It plays a crucial role in the proper functioning of an organism, and in terms of this work, particularly in the formation of a functional nervous system and maintaining its neuroplasticity [44]. Importantly, during the neurodevelopmental phase, neurons utilize apoptosis-related pathways for non-detrimental processes, such as axon pruning and axonal outgrowth [45]. Our previous research described molecular mechanisms induced by BP-3 in neurons, with a particular emphasis on the developing mammalian brain. Our recent in vitro setups involved both (1) primary cultures of mice neuronal cells treated with BP-3 and (2) primary cultures of mice neuronal cells settled from embryos prenatally exposed to BP-3. These studies showed the increase in neurotoxicity and apoptosis induced by BP-3, along with the downregulation of autophagy [32,46,47,48]. In the context of the nervous system, apoptosis is particularly important during neurogenesis, when 50% of neuronal cells are eliminated in the course of this process; apoptosis is thus crucial for the emergence of a correctly formed nervous system in a developed organism [49]. Therefore, determining the role of cerebral apoptosis in multigenerational BP-3-induced cell death may be the basis for predicting the prevalence of various types of neurodegenerative and neurodevelopmental disorders in adult life. In order to distinguish different kinds of multigenerational effects, we differentiate between intergenerational effects, describing the impact of the exposure of the paternal (P) generation on their immediate offspring (P→F_1_), and transgenerational effects, the impact exerted on subsequent generations (P→F_2_).

Prenatal exposure to a substance such as BP-3 may be the basis for the development of disorders like autism and schizophrenia, that will manifest themselves only in the adult nervous system [50,51,52]. Although studies on effects of BP-3 in this regard remain scarce, this speculation is heavily supported by research on other environmental contaminants, mainly other endocrine disruptors [53,54,55,56,57,58,59,60]. Despite evidence of the presence of BP-3 in human tissues, little is known about the effect of this substance on the nervous system, especially in the early stages of brain development.

Therefore, the aim of the current study was to investigate effects of prenatal exposure to BP-3 in the cerebral cortex and hippocampus of brains derived from F_1_ (in the offspring of mothers exposed to environmentally relevant concentrations of BP-3) and F_2_ mice (second-generation mice). In the present study, we focused on two brain structures crucially involved in neurodevelopmental and neurodegenerative disorders, i.e., the cerebral cortex and the hippocampus. Developmental disturbances in these regions can result in lasting neurological and behavioral impairments [61,62]. The existing literature highlights how specific chemicals can disrupt the brain’s normal development, leading to long-term cognitive and behavioral issues [63]. The brain cortex is involved in higher-order functions, such as perception, reasoning, and decision-making, whereas the hippocampus is integral to learning and memory, playing a vital role in episodic memory formation. Both structures undergo significant development during the prenatal and early postnatal periods, resulting in the particular vulnerability of individuals at those stages to exposure to environmental chemicals. Such exposure, particularly to endocrine-disrupting chemicals (EDCs), may result in neuropathologies such as autism spectrum disorder (ASD) or attention deficit hyperactivity disorder (ADHD) [64]. Analysis of intergenerational and transgenerational consequences of BP-3 exposure furthers our understanding of the mechanisms of environmental chemicals with neurotoxic effects and their impacts on neurodevelopmental disorders’ onsets in later stages of life.

## 2. Materials and Methods

### 2.1. Materials

A Lactate Dehydrogenase Activity (LDH) Assay Kit, Peroxide Assay Kit, Caspase 3 and 8 Assay Kit, BP-3 (C_14_H_12_O_3_, molecular weight: 228.2433, CAS no. 131-57-7), peanut oil and Tween 20 were provided by Sigma-Aldrich (St. Louis, MO, USA). Mini-PROTEAN TGX precast gels (7.5%), Sodium dodecyl sulfate (SDS), a Bradford reagent and Laemmli sample buffer were purchased from Bio-Rad Laboratories (Hercules, CA, USA). The 2-mercaptoethanol was obtained from Carl Roth GmbH + Co. KG (Karlsruhe, Germany). The Fast Probe qPCR Master Mix and Protein Markers were purchased from EURx (Gdańsk, Poland). The High-Capacity cDNA reverse transcription kit, radioimmunoprecipitation assay (RIPA) lysis buffer and TaqMan probes for the apoptosis-related genes; *Fas* (Mm00433237_m1), *Fasl* (Mm00438864_m1), *Bax* (Mm00432051_m1), *Bcl2* (Mm00477631_m1), *Gsk3b* (Mm00444911_m1), and the reference genes; *Hprt* (Mm01318741_m1), *Actb* (Mm00607939_s1), and *Gapdh* (Mm05724508_g1) were obtained from Thermo Fisher Scientific (Waltham, MA, USA). Polyvinylidene difluoride (PVDF) membranes were obtained from Merck Millipore (Billerica, MA, USA). BM chemiluminescence blotting substrate was obtained from Roche Diagnostics GmbH (Mannheim, Germany). The RNeasy Mini Kit and QIAzol Lysis Reagent were obtained from Qiagen (Hilden, Germany). Anti-mouse antibody conjugated to horseradish peroxidase (sc-516102), donkey anti-goat antibody conjugated to horseradish peroxidase (sc-2020), anti-ACTB mouse monoclonal antibody (sc-47778), anti-GSK3β rabbit polyclonal antibody (sc-9166), anti-BCL2 mouse monoclonal antibody (sc-7382), anti-BAX mouse monoclonal antibody (sc-7480), anti-FAS mouse monoclonal antibody (sc-74540), and anti-FASL mouse monoclonal antibody (sc-19681) were purchased from Santa Cruz Biotechnology Inc. (Santa Cruz, CA, USA). All other chemicals were of analytical or laboratory grade and were purchased from standard suppliers.

### 2.2. Animals and Treatment

**Scheme 1:**
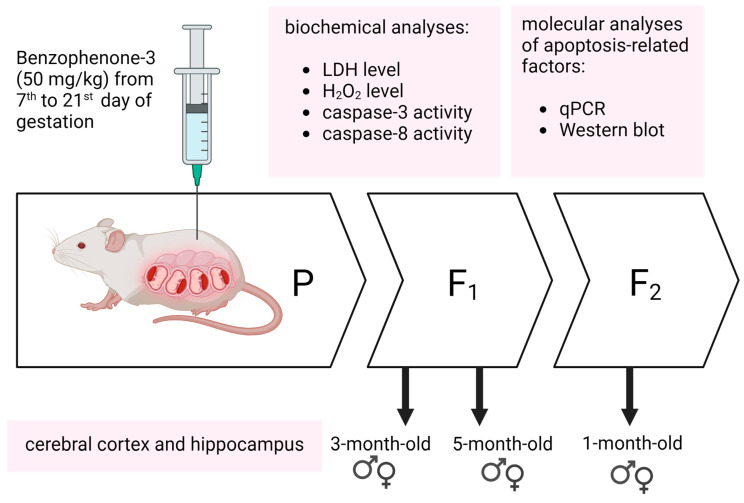
**Illustration of experimental paradigm**–prenatal exposure to BP-3 and analyses performed on the F_1_ and F_2_ generations to determine the multigenerational neurotoxicity and apoptosis induced by BP-3.

In our study, all pregnant Albino Swiss mice (Charles River Laboratories, Sulzfeld, Germany) were housed individually in cages with free access to water and food, and under environmentally controlled conditions (21 ± 1 °C; 40–50% humidity; 12:12 light:dark cycle). All animals were handled daily before the beginning of the experiments. The pregnant mice were treated with peanut oil (control group, 6 mice) or BP-3 (experimental group, 9 mice) by subcutaneous injections, administered from the 7th day of gestation until the 21st day (Scheme 1). BP-3 was dissolved in peanut oil and the animals were injected in a volume of 10 mL per kg of body weight, receiving a dose of 50 mg/kg daily. The selected dose of BP-3 was environmentally relevant, since it corresponded with the exposure determined for an average female (weighing from 50.3 kg to 99.2 kg, with a mean value of 75 kg according to the NCD [65] in the course of a full-body topical application of sunscreen—40 g per person, which translates to 2 mg per cm^2^ of skin). A singular application of sunscreen consists 4 g of BP-3, which translates to a dose of 53.33 mg/kg of substance per kg of body mass of an average female [66]. According to Wnuk et al. [48], exposure to BP-3 in the aforementioned form did not induce visible side effects in pregnant mice and did not affect the course of pregnancy. After the animals from the F_1_ generation reached sexual maturity, one group of the animals (3-month-old–20 males and 32 females, and 5-month-old–30 males and 43 females) were sacrificed to collect tissues, and the other group was used for crossbreeding to obtain the F_2_ generation. In order to avoid inbreeding, animals from different litters were used for obtaining the F_2_ generation. Mice from the F_2_ generation (1-month-old animals, 50 males and 49 females) were sacrificed to obtain brain tissues, as previously described [67]. The cortices and hippocampi were isolated from both the males and females. All efforts were made to minimize the suffering and the number of animals in accordance with the National Institutes of Health Guidelines for the Care and Use of Laboratory Animals and the European Communities Council Directive for the Care and Use of Laboratory Animals (86/609/EEC). Experimental protocols were approved by the Committee for Laboratory Animal Welfare and the Ethics Committee of the Maj Institute of Pharmacology PAS in Krakow, Poland, resolution no 137/2019.

### 2.3. Assessment of Lactate Dehydrogenase (LDH) Level

LDH is an oxidoreductase enzyme catalyzing a bidirectional conversion of lactate and pyruvate, and the assessment of its activity is a widely used marker of tissue damage. In this study, the levels of LDH were assessed with an LDH Assay Kit on a 96-well plate, according to protocol provided by manufacturer. The isolated structures were first homogenized in RIPA buffer and centrifuged (10,000× *g* for 15 min in 4 °C) in order to separate the insoluble elements. The final reaction volume consisted of 50 µL of a hippocampus or cerebral cortex sample and 50 µL of a master reaction mix, and the protein concentration was set to be the same in each sample. The absorbance measurement was conducted at a wavelength of 450 nm using an Infinite M200PRO microplate reader (Tecan, Männedorf, Switzerland). The data were analyzed with i-control 1.11 software and are presented as a percentage of the control value ± SEM.

### 2.4. Assessment of Hydrogen Peroxide (H_2_O_2_) Concentration

H_2_O_2_ arises in the course of molecular oxygen metabolism and serves as a messenger taking part in intracellular signaling, as well as a source of oxidative stress. The quantitative determination of the hydrogen peroxide in a hippocampus or cerebral cortex sample is possible due to the formation of the colorimetric purple complex in response to the oxidation of Fe^2+^ to Fe^3+^ by peroxides. In our study, the concentration of H_2_O_2_ was measured with a Peroxide Assay Kit on a standard 96-well plate, according to a protocol provided by the manufacturer. The tissue lysates originating from the hippocampus or cerebral cortex were incubated with 200 µL of detection reagent for 30 min at room temperature. Next, the absorbance was measured with an Infinite M200PRO microplate reader and a wavelength of 585 nm. The intensity of the reaction color was proportional to the peroxide concentration in the sample. The obtained results are presented as a percentage of the control value ± SEM.

### 2.5. Assessment of Caspase-8 Activity

The role of caspase-8 is the activation of caspases-3, -6, and -7, making it an important factor in apoptotic processes. Caspase-8 activity was measured using the Caspase-8 Assay Kit (Sigma Aldrich, St. Louis, MO, USA) according to the manufacturer’s instruction. The colorimetric assessment is based on measuring the hydrolysis of the peptide substrate Ac-IETD-*p*NA (Acetyl-Ile-Glu-Thr-Asp *p*-Nitroanilide) and the release of *p*-nitroanilide moieties (*p*NA) that exhibit high levels of absorption at a wavelength of 405 nm. The assessment was conducted on a standard 96-well plate, with a positive and negative control. Subsequent wells were filled with 10 µL of a hippocampus or cerebral cortex sample and a corresponding amount of 1× Assay Buffer. The reaction was initialized by adding caspase-8 colorimetric substrate using a multichannel pipette. The absorbance was measured at a wavelength of 405 nm. The data were analyzed with i-control 1.11 software and the results are presented as a percentage of the control value ± SEM.

### 2.6. Assessment of Caspase-3 Activity

In our study, the activity of caspase-3, an effector caspase during apoptotic processes, was assessed with the use of the Caspase 3 Assay Kit (Sigma Aldrich, USA) in accordance with the manufacturer’s protocol. This colorimetric assay was based on measuring the hydrolysis of the chromogenic substrate of caspase-3 (Ac-DEVD-*p*NA; Acetyl-ASP-Glu-Val-Asp *p*-nitroanilide) and the release of *p*-nitroanilide moieties (*p*NA) that were detected as the change in the absorbance value at a wavelength of 405 nm. The assessment was performed in a 100 µL volume consisting of a hippocampus or cerebral cortex sample, the 1× Assay Buffer, and the caspase-3 substrate. After incubation, the absorbance was measured at a wavelength of 405 nm using an Infinite M200PRO microplate reader and the results are presented as a percentage of the control value  ±  SEM.

### 2.7. qPCR Analysis of the Apoptosis-Related Factors

Mouse cerebral cortices and hippocampi were lysed using the QIAzol Lysis Reagent and the total RNA was isolated with the RNeasy Mini Kit, as previously described [68]. The amount of extracted RNA was quantified spectrophotometrically at 260 nm and 260/280 nm, with the accepted purity of RNA determined at an A260/A280 ratio value of ~2.0. To generate the cDNA, the RNA template was reverse transcribed with a High-Capacity cDNA Reverse Transcription Kit using the CFX96 Real-Time system (Bio-Rad, USA), in accordance with manufacturer’s protocol. The cDNA obtained from the reaction was amplified during the qPCR procedure consisting of the following steps: 2 min at 50 °C and 10 min at 95 °C, followed by 40 cycles of 15 s at 95 °C and 1 min at 60 °C. In addition to cDNA, the reaction mixture was composed of Fast Probe qPCR Master Mix, the TaqMan Gene Expression Assay (specific for *Gapdh, Hprt, Actb, Fas*, *Fasl*, *Bax*, *Bcl2*, and *Gsk3b*) and RNase-free water. The qPCR was performed with the CFX96 Real-Time system and the data were analyzed using the delta delta Ct method. The reference genes were selected from *Gapdh, Hprt*, and *Actb* with the use of the following algorithms: geNorm, NormFinder, and BestKeeper. A full analysis of candidates for reference genes is available in the Supplementary Information (SI 1). The results are presented as fold change ± SEM. TaqMan probes targeting apoptosis-related genes and reference genes used in this study are presented in Table 1.

### 2.8. Western Blot Analysis of the Apoptosis-Related Factors

After the isolation of the cerebral cortex and hippocampus, the structures underwent mechanical homogenization and sonication in the RIPA lysing buffer. Subsequently, the homogenate was centrifuged at 15,000× *g* and 4 °C for 20 min. The protein concentration was quantified using the Bradford reagent and bovine serum albumin as a standard. Next, the Laemmli buffer was added along with β-mercaptoethanol, and the samples were subjected to denaturation on a block heater for 5 min at 95 °C. Samples were then separated using 7.5% polyacrylamide gels and TriColor or Rainbow markers. After electrophoresis, the separated proteins were electrotransferred onto a PVDF membrane with the Bio-Rad Mini Trans-Blot apparatus. To reduce non-specific signals, the membranes were washed with 5% dried milk and 0.2% Tween-20 in 0.02M Tris-buffered saline (TBS) for two hours. Subsequently, the membranes were incubated overnight at 4 °C with one of the primary antibodies targeting the reference protein ACTB or the studied proteins: FAS, FASL, BAX, BCL2, and GSK3β. Antibodies used in this research and their appropriate dilutions are as follows: anti-ACTB mouse monoclonal antibody (1:3500), anti-GSK3β rabbit polyclonal antibody (1:150), anti-BCL2 mouse monoclonal antibody (1:100), anti-BAX mouse monoclonal antibody (1:100), anti-FAS mouse monoclonal antibody (1:100), and anti-FASL mouse monoclonal antibody (1:100). The next day, the membranes were washed and incubated with secondary antibodies (diluted 1:1000) conjugated with horseradish peroxidase (HRP) to detect the target to which the primary antibody was bound. The chemiluminescence band signals were developed using the BM Chemiluminescence Blotting Substrate and visualized with Luminescent Image Analyzer Fuji-Las 4000 (Fuji, Japan). Luminescence intensity was quantified with a MultiGauge V3.0 image analyser and normalized to ACTB, followed by presenting the results as a percentage of the control value ± SEM. Antibodies targeting the apoptosis-related protein and reference protein used in this study are presented in Table 2.

### 2.9. Data Analysis

The statistical analysis was accomplished with the use of raw data expressed in the following ways: mean absorbance intensity (in arbitrary units) per sample for analyses of LDH, H_2_O_2_, caspase-3 and -8 activities; fluorescence units for qPCR; and mean optical density for Western blot. Each control or experimental group consisted of animals from different litters and the number of animals per group was 5–6. The analyses were preceded by a Shapiro–Wilk normality test. Student’s *t*-test was applied to assess the differences between the control (peanut oil injections) and experimental groups (BP-3 injections). The differences were considered significant when *p* values reached less than 0.05 and were indicated as follows: * *p* < 0.05; ** *p* < 0.01; and *** *p* < 0.001 (versus the control mice).

## 3. Results

### 3.1. In 3-Month-Old Offspring, Prenatal Exposure to BP-3 Elevates LDH and H_2_O_2_ Levels in Female Hippocampi, but Decreases Caspase-3 Activity in Female Cerebral Cortices

In our experiment, pregnant female mice were exposed to BP-3 in concentrations of 50 mg/kg between the 7th and 21st day of pregnancy. We aimed to assess the effects of prenatal exposure to BP-3 in 3-month-old (adolescent) and 5-month-old (adult) offspring (F_1_) and transgenerational changes in 1-month-old individuals (F_2_). We chose to study intergenerational/transgenerational effects of BP-3 on two brain structures, i.e., the cerebral cortex and the hippocampus, since they are known for their crucial involvement in developmental and degenerative brain disorders [61,62,63,64].

**Figure 1:**
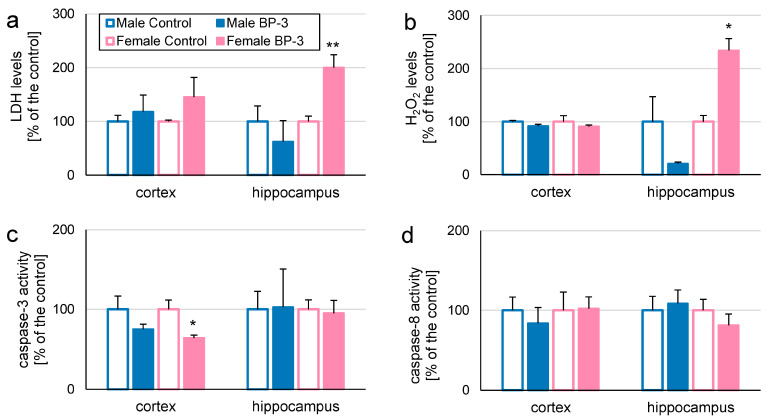
**The effects of prenatal exposure to BP-3 in 3-month-old mice from the F_1_ generation on levels of LDH, H_2_O_2_, and activity of caspase-3 and -8.** Prenatal exposure to BP-3 increased LDH (**a**) and H_2_O_2_ levels (**b**) in female hippocampi and decreased caspase-3 activity in female cerebral cortices (**c**) in 3-month-old F_1_ mice. In the case of caspase-8, there were no significant changes in its activity in female and male cerebral cortices or hippocampi (**d**). Each control or experimental group consisted of animals from different litters, and the samples were collected from 5 to 6 animals per group. All results are expressed as percentages of the control ± SEM. * *p* < 0.05 and ** *p* < 0.01 versus the control animals.

Prenatal exposure to BP-3 caused pronounced changes in levels of LDH and H_2_O_2_ in the hippocampi of adolescent (3-month-old) female offspring (200 and 234% of the control, respectively; Figure 1a,b). Furthermore, in the cerebral cortices of 3-month-old female offspring, we observed decreased caspase-3 activity (64% of the control; Figure 1c). We noticed no biochemical changes in the cerebral cortex, nor in the hippocampus, in 3-month-old male offspring (Figure 1a–d). In females, cortical LDH, H_2_O_2_ and caspase-8 levels as well as hippocampal caspase-3 and caspase-8 levels were not affected (Figure 1a–d).

### 3.2. Prenatal Exposure to BP-3 Causes Pronounced Changes in the Expression of Apoptosis-Related Factors in Male Cerebral Cortices and Hippocampi, and Female Hippocampi of 3-Month-Old Offspring

**Figure 2:**
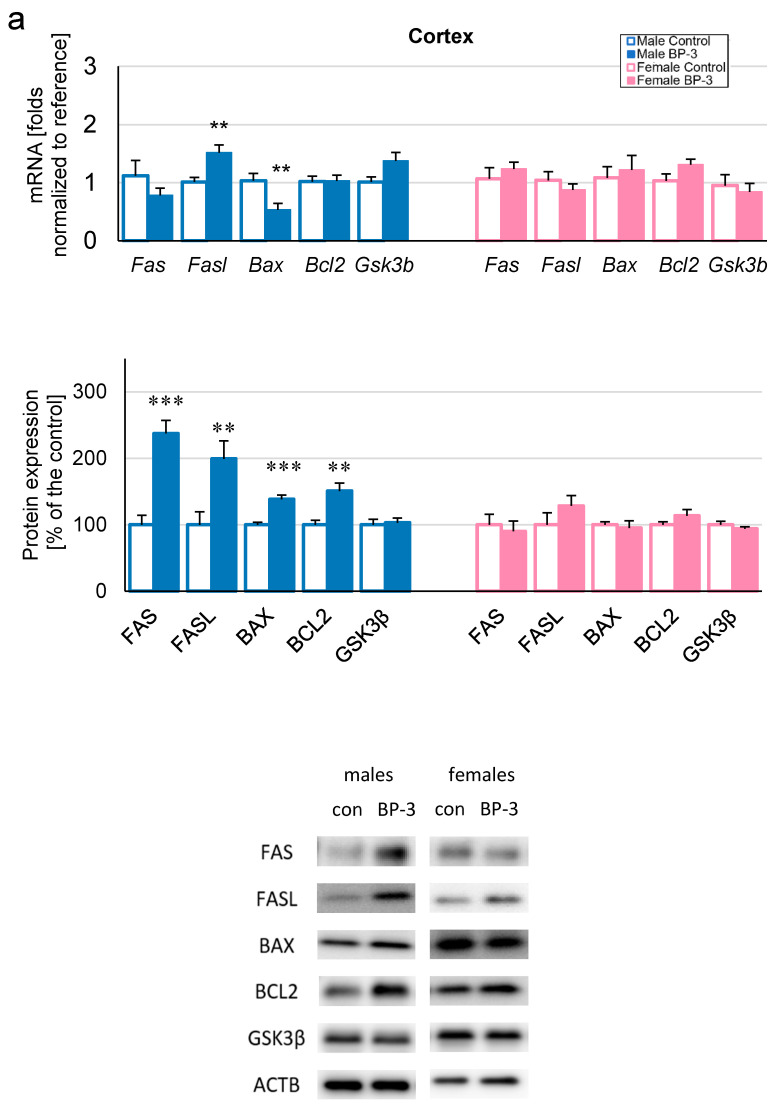

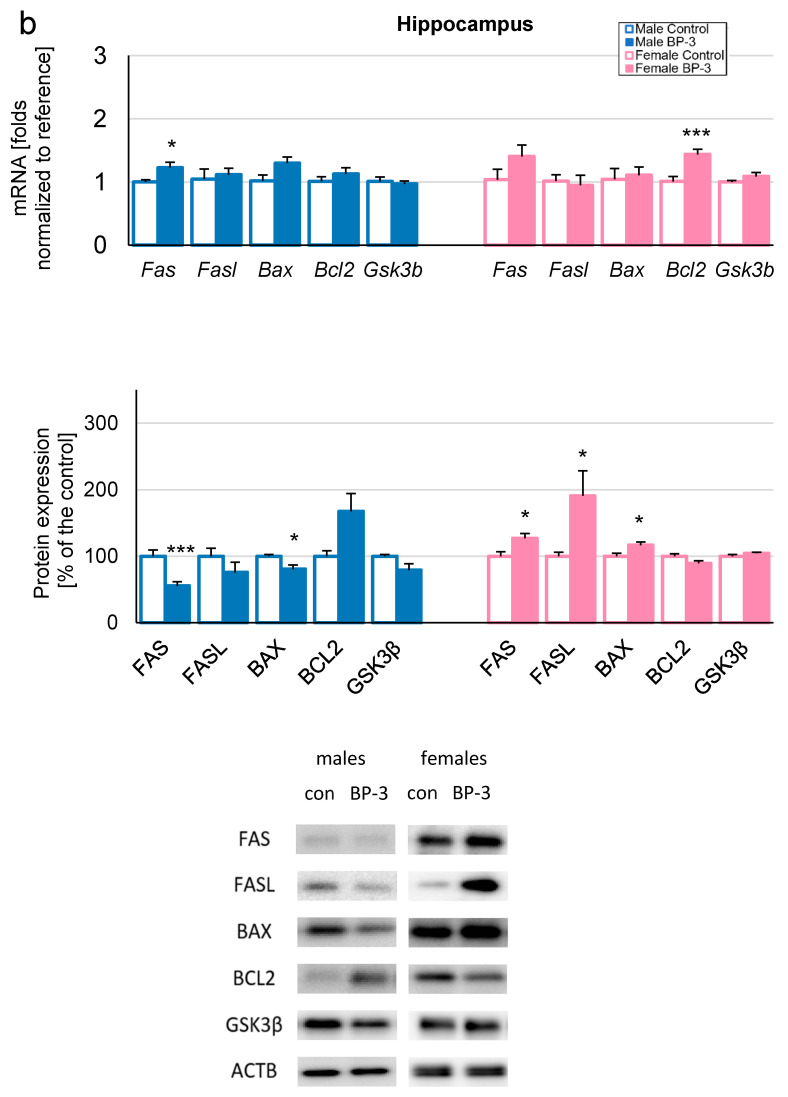
**The effects of prenatal exposure to BP-3 in 3-month-old mice from the F_1_ generation on the gene and protein expression of apoptosis-related factors.** Prenatal exposure to BP-3 diminished the mRNA of *Bax*, but enhanced levels of FAS, *Fasl*/FASL, BAX, and BCL2 in 3-month-old F_1_ males cortices; however, there were no expression changes in apoptosis-related factors in the cerebral cortices of 3-month-old F_1_ females (**a**). For the hippocampi, prenatal exposure to BP-3 increased the mRNA expression of *Fas*, but decreased FAS and BAX in 3-month-old F_1_ males. Prenatal exposure of 3-month-old F_1_ females to BP-3 resulted in an increase in *Bcl2* mRNA, as well as FAS, FASL, BAX protein levels in hippocampi (**b**). Each control or experimental group consisted of animals from different litters, and the samples were collected from 5 to 6 animals per group. All results are expressed as a fold change in the case of qPCR and a percentage of the control ± SEM in the case of Western blotting. * *p* < 0.05, ** *p* < 0.01, and *** *p* < 0.001 versus the control animals.

Apoptosis plays a crucial role in neurodevelopment and neurodegeneration. This fact prompted us to investigate the intergenerational/transgenerational effects of BP-3 on apoptosis-related factors in the chosen brain regions (cerebral cortex and hippocampus). In 3-month-old offspring, dysregulation of the apoptosis-related mRNAs and protein was most prominent in the cerebral cortices of males (Figure 2a), where prenatal exposure to BP-3 caused the upregulation of FAS (237% of the control), *Fasl* (to 1.52 fold)/FASL (199% of the control), BAX (138% of the control) and BCL2 (151% of the control), but the downregulation of *Bax* (to 0.53 fold). In contrast, no changes were observed in the cerebral cortices of females (Figure 2a).

In the hippocampi of 3-month-old males, prenatal exposure to BP-3 increased the mRNA of *Fas* (1.23 fold) but decreased the FAS protein level (55% of the control), while also causing BAX downregulation (81% of the control) (Figure 2b). The levels of remaining factors (*Fasl*/FASL, *Bax*, *Bcl2*/BCL2, and *Gsk3b*/GSK3β) were not affected. In the hippocampi of 3-month-old females, *Bcl2* (1.44 fold), FAS (127% of the control), FASL (191% of the control), and BAX (117% of the control) were upregulated, while the rest of the investigated factors (*Fas*, *Fasl*, *Bax*, BCL2, and *Gsk3b*/GSK3β) remained unchanged (Figure 2b).

### 3.3. Prenatal Exposure to BP-3 Alters H_2_O_2_ Levels and Caspase-8 Activity in Cerebral Cortices and/or Hippocampi of 5-Month-Old Offspring

**Figure 3:**
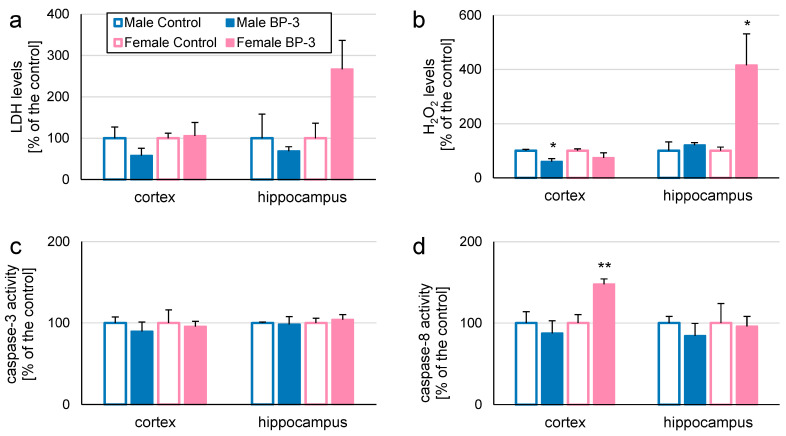
**The effects of prenatal exposure to BP-3 in 5-month-old mice from the F_1_ generation on levels of LDH, H_2_O_2_, and the activity of caspase-3 and -8.** Prenatal exposure of 5-month-old F_1_ males reduced H_2_O_2_ levels in the cerebral cortex (**b**); while for females, significant changes concerned only an increase in caspase-8 activity in the cerebral cortex (**d**) and increases in H_2_O_2_ levels in the hippocampus (**b**). No changes were noticed in parameters determining LDH levels (**a**) and caspase-3 activity (**c**) in 5-month-old F_1_ males and females. Each control or experimental group consisted of animals from different litters, and the samples were collected from 5 to 6 animals per group. All results are expressed as a percentage of the control ± SEM. * *p* < 0.05 and ** *p* < 0.01 versus the control animals.

The most pronounced effects of prenatal exposure to BP-3 in 5-month-old offspring were noticed in H_2_O_2_ levels in the female hippocampus (416% of the control) but not in the cerebral cortices of females (Figure 3b). In contrast, no changes in this parameter were present in the hippocampi of males, while decreased H_2_O_2_ levels were observed in cerebral cortices (60% of the control; Figure 3b). In 5-month-old offspring, prenatal exposure to BP-3 altered caspase-8 only in the cerebral cortices of females, increasing its activity to 148% of the control (Figure 3d). No changes in LDH levels and caspase-3 activity were observed in these groups in either the cerebral cortex or the hippocampus (Figure 3a,c). 

### 3.4. Prenatal Exposure to BP-3 Changes Expression of Apoptosis-Related Factors in Cerebral Cortices and/or Hippocampi of 5-Month-Old Offspring

**Figure 4:**
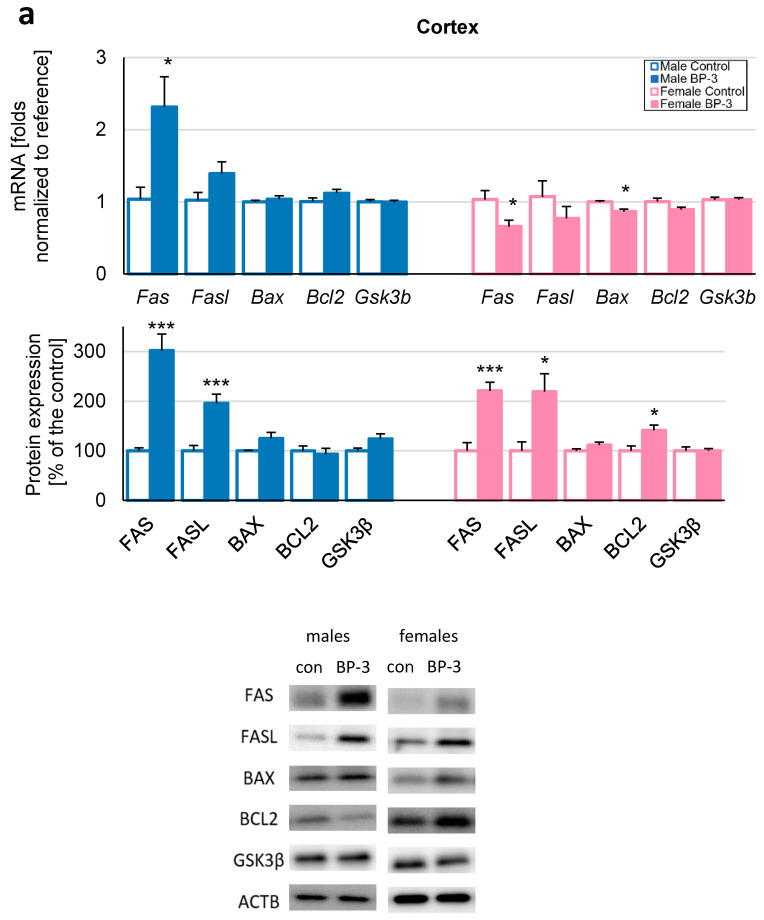

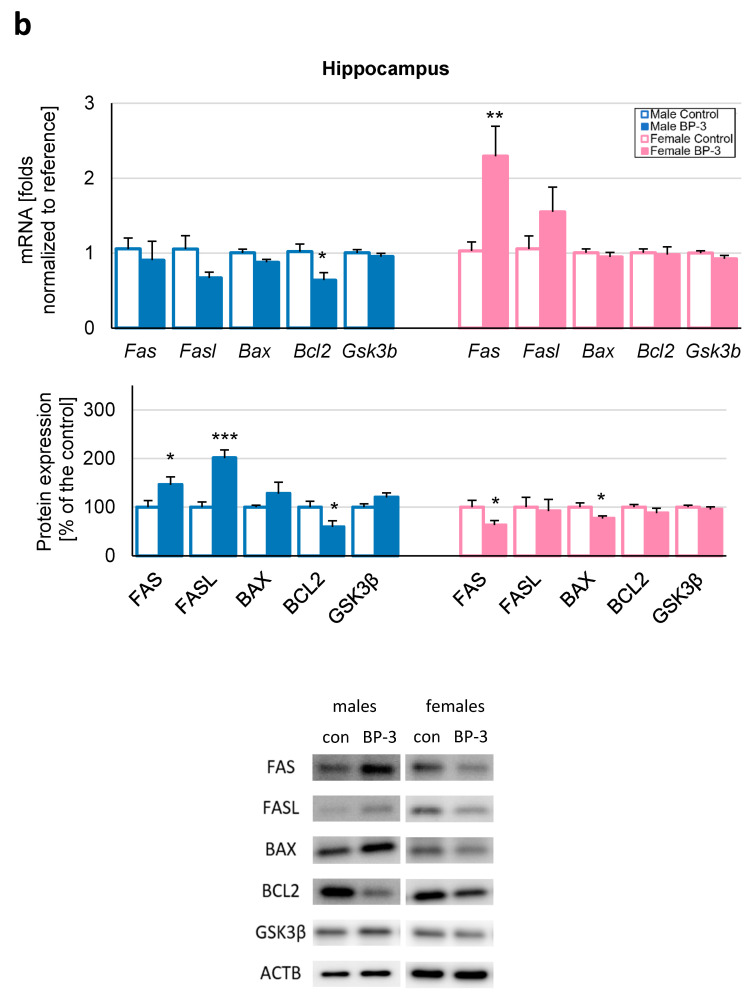
**The effects of prenatal exposure to BP-3 in 5-month-old mice from the F_1_ generation on the gene and protein expression of apoptosis-related factors.** Prenatal exposure to BP-3 increased *Fas*/FAS and FASL mRNA or protein levels in the cerebral cortices of 5-month-old males, while in females, there were decreases in mRNA *Fas* and *Bax* expression and enhancements in FAS, FASL, and BCL2 protein levels (**a**). Within the hippocampus, significant changes included a *Bcl2*/BCL2 decrease and FAS, FASL increases in 5-month-old males; in females, significant changes included an enhancement in *Fas* mRNA expression and diminishment in FAS and BAX protein levels (**b**). Each control or experimental group consisted of animals from different litters, and the samples were collected from 5 to 6 animals per group. All results are expressed as a fold change in the case of qPCR and a percentage of the control ± SEM in the case of Western blotting. * *p* < 0.05, ** *p* < 0.01, and *** *p* < 0.001 versus the control animals.

In the cerebral cortices of 5-month-old male offspring prenatally exposed to BP-3, increased expressions of *Fas* (2.31-fold increase), FAS (302% of the control), and FASL (196% of the control) were observed. In females, prenatal exposure to BP-3 caused a decrease in *Fas* (to 0.66-fold) at an mRNA level, but increases at the protein level of FAS (221% of the control) and FASL (219% of the control). In the cerebral cortices of 5-month-old offspring, prenatal exposure to BP-3 did not alter *Bax*/BAX, *Bcl2*/BCL2, and *Gsk3b*/GSK3β, except for the mRNA of *Bax* (decrease to 0.87-fold) and BCL2 in females (increased to 141% of the control; Figure 4a). 

In the hippocampi of 5-month-old male offspring prenatally exposed to BP-3, we observed increased FAS (160% of the control) and FASL (201% of the control) levels and a decrease in *Bcl2*/BCL2 (to 0.64 fold and 59% of the control, respectively). In females, there is a discrepancy in effects of prenatal exposure to BP-3 in *Fas*/FAS mRNA and protein levels, i.e., *Fas* increased 2.29-fold and FAS decreased to 63% of the control. In female offspring, BP-3 also caused the BAX level decrease (71% of the control; Figure 4b). Levels of remaining the apoptosis-related mRNAs and protein did not change (Figure 4a,b).

### 3.5. Transgenerational Effects of Exposure to BP-3 Alters LDH, Caspase-3, and Caspase-8 Activity in Cerebral Cortices and/or Hippocampi of 1-Month-Old Mice

**Figure 5:**
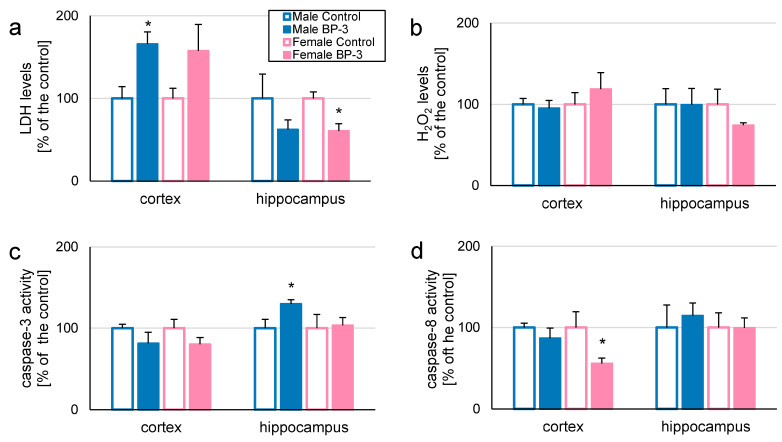
**The effects of BP-3 in 1-month-old mice from the F_2_ generation on levels of LDH, H_2_O_2_, and the activity of caspase-3 and -8.** Ancestral exposure of gestating mice to BP-3 resulted in increases in LDH levels in the cerebral cortices (**a**) and caspase-3 activity in the hippocampi (**c**) of 1-month-old F_2_ males. In females, the significant changes included decreases in caspase-8 activity in the cerebral cortex (**d**) and LDH levels in the hippocampus (**a**). The levels of H_2_O_2_ were not influenced (**b**). Each control or experimental group consisted of animals from different litters, and the samples were collected from 5 to 6 animals per group. All results are expressed as a percentage of the control ± SEM. * *p* < 0.05 versus the control animals.

In 1-month-old males from the F_2_ generation, transgenerational effects of exposure to BP-3 increased LDH levels in the cerebral cortex (166% of the control), but not in the hippocampus and elevated caspase-3 activity in the hippocampus (130% of the control), but not in the cerebral cortex (Figure 5a,c). On the other hand, in females, transgenerational effects of exposure to BP-3 decreased LDH levels in the hippocampus (61% of the control), but not in the cerebral cortex, and weakened caspase-8 activity in the cerebral cortex (56% of the control), but not in the hippocampus (Figure 5a,d). F_2_ individuals were not affected by transgenerational effects of exposure to BP-3 in terms of H_2_O_2_ levels (Figure 5b). 

### 3.6. Transgenerational Effects of Exposure to BP-3 Changes Expression of Apoptosis-Related Factors in Cerebral Cortices and/or Hippocampi of 1-Month-Old Individuals

**Figure 6:**
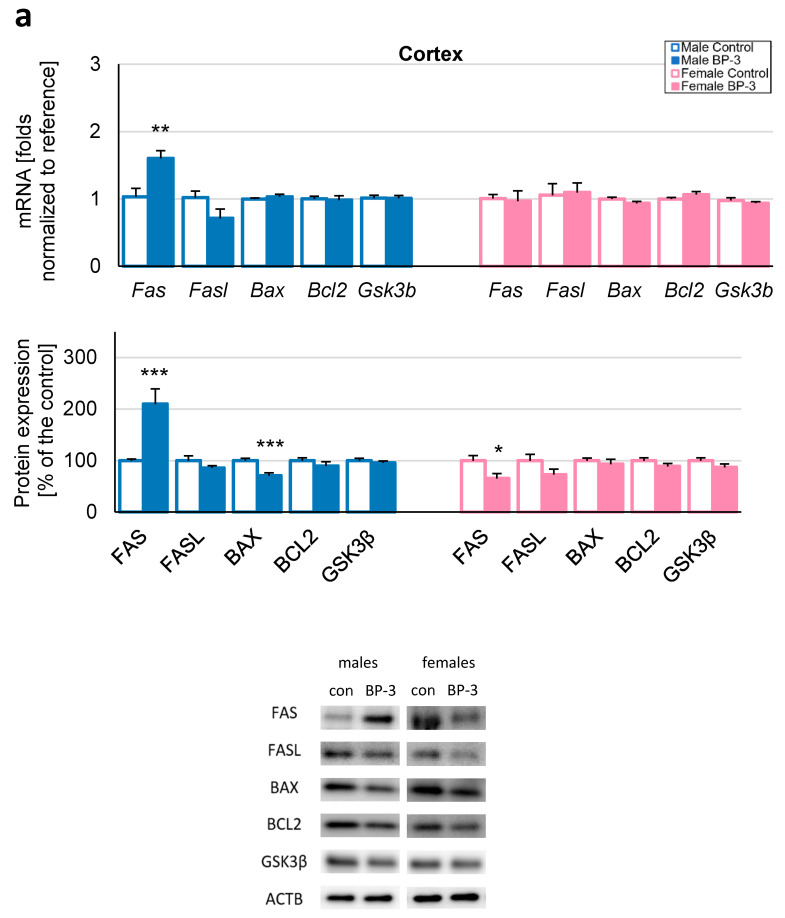

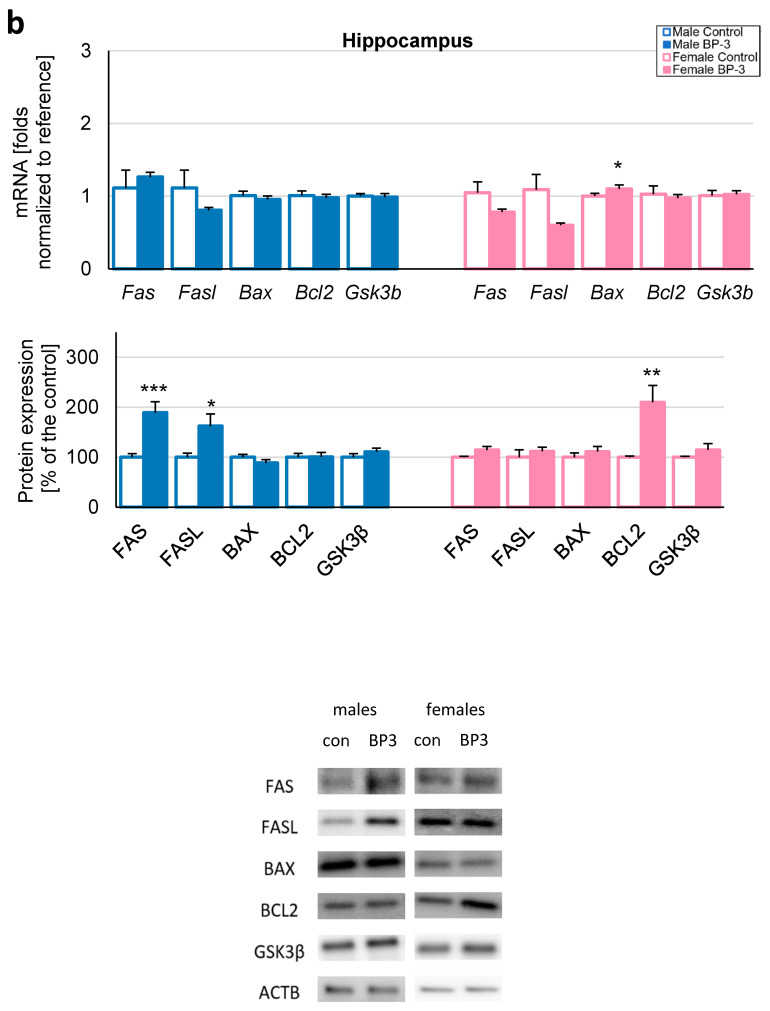
**The effects of BP-3 in 1-month-old mice from the F_2_ generation on the gene and protein expression of apoptosis-related factors.** Ancestral exposure of gestating mice to BP-3 resulted in the enhancement of *Fas*/FAS and the diminishment of BAX expression in cerebral cortices of 1-month-old F_2_ males, while in females, only a decrease in the FAS level (**a**) was observed. Within the hippocampus, significant changes included an increase in FAS and FASL protein levels in 1-month-old F_2_ males; in females, the significant changes included the increase in the *Bax* mRNA expression level and in the BCL2 protein level (**b**). Each control or experimental group consisted of animals from different litters, and the samples were collected from 5 to 6 animals per group. All results are expressed as a fold change in the case of qPCR and as a percentage of the control ± SEM in the case of Western blotting. * *p* < 0.05, ** *p* < 0.01, and *** *p* < 0.001 versus the control animals.

In the cerebral cortices of 1-month-old individuals from the F_2_ generation, transgenerational effects of exposure to BP-3 increased *Fas* and FAS (1.60 fold and 210% of the control, respectively), but decreased BAX (71% of the control) in males, while in females, FAS decreased to 66% of the control (Figure 6a). 

In the hippocampi of 1-month-old individuals, FAS and FASL were elevated in males (189 and 177% of the control, respectively), while in females, an increase in *Bax* (1.10 fold) and BCL2 was observed (210% of the control; Figure 6b). Levels of the remaining apoptosis-related mRNAs and protein did not change (Figure 6a,b).

## 4. Discussion

BP-3 is widely applied as a UV filter in personal care products and as a stabilizer for commercial use that prevents photodegradation. Emerging evidence suggests that BP-3 may act as an endocrine disruptor, interfering with the body’s endocrine system. Although studies showed that BP-3 crosses the BBB and its presence was detected in *post mortem* adult brains, the mechanisms of BP-3 action in the central nervous system are still an important subject of interest for researchers [31]. In our recent in vitro studies, we conducted experiments on primary cultures of mice neuronal cells treated with BP-3, and primary neuronal cells settled from embryos prenatally exposed to BP-3. We demonstrated that BP-3 induced neurotoxicity and apoptosis, and downregulated autophagy [32,46,47,48], suggesting its contribution to neurodevelopmental abnormalities and the onset of neurological disorders. However, little is known about its action on neurodevelopment, especially in the contexts of intergenerational and transgenerational consequences. Therefore, the aim of our study was to determine the toxic and apoptotic effects of prenatal exposure to BP-3 in mice from the F_1_ and F_2_ generations, both in males and females. The administered dose of BP-3 to the parental generation was relevant to the amount that an average woman is exposed to as a result of applying sunscreen to the entire body (40 g of cream/person − 2 mg/cm^2^ of skin) [65,66,69].

Here, we provide evidence for the neurotoxic action of BP-3 on the mouse cerebral cortex and hippocampus in terms of LDH, caspase-3, caspase-8 and/or H_2_O_2_ levels in the offspring of mothers exposed to environmentally relevant concentrations of BP-3, as well as in the second-generation mice. Sensitivity to BP-3, as regards the levels of LDH and caspase-3 activity, was observed in both generations (F_1_ and F_2_), whereas sensitivity to BP-3 regarding H_2_O_2_ levels was observed only in the F_1_ generation. Most vulnerable to LDH- and H_2_O_2_-related effects was the female hippocampus, whereas vulnerability to caspase-3-related effects was only observed in the female cerebral cortex (F_1_ generation) and the male hippocampus (F_2_ generation). These data show that BP-3 evokes a variety of effects which may either initiate apoptosis (caspase-3), possible via the induction of oxidative stress (H_2_O_2_), and finally evoke brain cell death (LDH). Increases in LDH and H_2_O_2_ release are related to the onset of neurodegenerative disorders [70,71], autism [72], schizophrenia, or social adaptation disorders [73] and similar results to ours were obtained in in vitro studies on primary neurons [46,47,48] or the SH-SY5Y cell line [74] exposed to BP-3. On the other hand, our data indicating decreases in LDH or H_2_O_2_ release may be associated with a cellular defense response aimed at delaying neurodegeneration [75]. The obtained results indicating increasing levels of caspase-3 activity are consistent with the results previously obtained in vitro [46,47,48]. Therefore, our present study shows that BP-3 may predispose brain tissue to caspase-3-dependent apoptosis and cause multigenerational neurotoxic effects.

The pro-apoptotic effect of BP-3 on the brain was confirmed by increases in *Fas*/FAS and/or *Fasl*/FASL expression levels that were independent from generation, gender, or cerebral structure, except for in 3-month-old F_1_ females and 1-month-old F_2_ females. In addition, BP-3 affected caspase-8 activity (known to participate in the FAS/FASL-dependent extrinsic apoptosis pathway) in both generations, but the only vulnerable structure was the female cerebral cortex. Enhanced FAS and FASL expression levels in brain cells accompany neurodegenerative and neurological disorders’ onsets, i.e., during experimental autoimmune encephalomyelitis (EAE model), exposure to heavy metals (e.g., cadmium), as well as in Alzheimer’s disease [76,77,78]. Our data indicated that BP-3 also caused the dysregulation of the BAX/BCL2 pathway which was much more prominent in the F_1_ than in the F_2_ generation. In addition, we observed the prevalence of extrinsic apoptosis (FAS, FASL, and caspase-8) over intrinsic apoptosis (BAX, BCL2) in both generations, except for in the 3-month-old F_1_ generation. Similarly to our studies, simultaneous increase in anti-apoptotic BCL2 and pro-apoptotic BAX was observed during prenatal exposure to cocaine [79] and cellular models of stroke and Alzheimer’s disease [80,81,82,83,84]. The anti-apoptotic properties of BCL2 are also prominent in the mechanism of cell protection against the effects of hypoxia and reperfusion, as evidenced by an enhanced BCL2 expression level and the increased survival of cerebral cortex cells in rats subjected to hypoxia [71]. Therefore, apoptotic processes induced by BP-3 were mostly related to the extrinsic pathway and these changes affected F_1_ and F_2_ males to a greater extent.

Taking into account the main goal of the research to demonstrate the transgenerational consequences of BP-3 exposure, it can be said that BP-3 has an ability to induce brain malfunction (due to neurotoxicity and apoptosis) and in this way, it may contribute to neurodevelopmental diseases in F_2_ generation mice. Considering gender differences, males appear to be more vulnerable than females to the impact of BP-3 exposure, as evidenced by the increase in LDH and *Fas*/FAS levels in the cerebral cortex or the enhancement in caspase-3 activity as well as FAS and FASL expression levels in the hippocampus. The fewer effects observed in females may be associated with the protective role of estrogens in the brain [85]. Males’ sensitivity to transgenerational action was also observed after exposure to vinclozolin, an agricultural fungicide, known to be an androgenic endocrine disruptor, that induced epigenetic changes and the transgenerational inheritance of neurodevelopmental disorders and abnormalities [86,87]. Therefore, our studies indicated that ancestral exposure of gestating mice to BP-3 induces toxic and pro-apoptotic effects in the brains of second-generation mice; however, mechanisms of inheritance or such long-term influences of this substance still must be specified. While considering the results achieved in the current study, it is also important to note its limitations; we have conducted ex vivo experiments including biochemical and molecular assessments, and we observed and studied effects evoked in this regard. The results obtained in the course of our work encourage further efforts; the next stage of our work is to include neurobehavioral assessment in order to determine if the effects observed ex vivo correspond with changes in animals’ behavior. Additionally, our study did not assess the effects of BP-3 exposure on the P generation, including factors such as maternal health, food consumption, and weight gain during pregnancy. These factors could influence intrauterine conditions and, consequently, developmental outcomes in the offspring. While our research focused on the indirect effects of BP-3 on the nervous system and its transgenerational impacts, future studies should incorporate these aspects to provide a more comprehensive understanding of BP-3′s effects on both parental and subsequent generations.

In summary, we proved for the first time the toxic and pro-apoptotic effects of prenatal exposure to BP-3 that are both intergenerational, i.e., in the offspring of mothers exposed to environmentally relevant concentrations of BP-3, as well as transgenerational, i.e., in second-generation mice. Our studies indicate that apoptotic processes induced by BP-3 in the central nervous system are more closely related to the extrinsic pathway than to the intrinsic mitochondrial pathway. Interestingly, taking into account expression levels of apoptotic factors, we observe much stronger effects of prenatal exposure to BP-3 in males and in the F_1_ generation. In our studies, we identified transgenerational mechanisms, which confirm the capacity of BP-3 to impair brain homeostasis and in this way increase the risk of multigenerational disorders associated with the disruption of neurodevelopment.

## Figures and Tables

**Table 1:** TaqMan probes targeting apoptosis-related genes and reference genes used in this study.

Gene Name	Catalog Number of TaqMan Probe
*Fas*	Mm00433237_m1
*Fasl*	Mm00438864_m1
*Bax*	Mm00432051_m1
*Bcl2*	Mm00477631_m1
*Gsk3b*	Mm00444911_m1
*Hprt*	Mm01318741_m1
*Actb*	Mm00607939_s1
*Gapdh*	Mm05724508_g1

**Table 2:** Antibodies targeting the apoptosis-related protein and reference protein used in this study.

Protein Name	Catalog Number of an Anti-Body
FAS	sc-74540
FASL	sc-19681
BAX	sc-7480
BCL2	sc-7382
GSK3β	sc-9166
ACTB	sc-47778

## Data Availability

The datasets generated during and/or analyzed during the current study are available from the corresponding author on reasonable request.

## Supplementary Materials

The following supporting information can be downloaded at: https://www.mdpi.com/article/10.3390/toxics12120906/s1.

## Abbreviations

*Actb—*β-actin; *Bax*/BAX—BCL2-associated X protein; BBB—blood–brain barrier; *Bcl2*/BCL2—B-cell lymphoma 2; BP-3—benzophenone-3; Ct—threshold cycle; F_1_—first-generation mice; F_2—_second-generation mice; *Fas*/FAS—cell surface death receptor; Fasl/FASL—FAS ligand; *Gapdh*—glyceraldehyde-3-phosphate dehydrogenase; *Gsk3b*/GSK3β—glycogen synthase kinase 3 beta; *Hprt1*—Hypoxanthine phosphoribosyltransferase 1; H_2_O_2_—hydrogen peroxide; LDH—lactate dehydrogenase; P—parental generation; UV—ultraviolet light; qPCR—quantitative polymerase chain reaction.
