# Supplementary material for: Multigenerational Consequences of Prenatal Exposure to Benzophenone-3 Demonstrate Sex- and Region-Dependent Neurotoxic and Pro-Apoptotic Effects in Mouse Brain
Source: Toxics. 2024 Dec 13;12(12):906. doi: 10.3390/toxics12120906 (PMC11728767; doi:10.3390/toxics12120906)

Supplementary information

SI 1. Full analysis of candidates for reference genes

	Generation	Age	Sex	Brain structure	Reference gene
1.	F_1	3 month-old	Males	Cortex	*Hprt*
2.	F_1	3 month-old	Males	Hippocampus	*Hprt*
3.	F_1	3 month-old	Females	Cortex	*Gapdh*
4.	F_1	3 month-old	Females	Hippocampus	*Actb*
5.	F_1	5 month-old	Males	Cortex	*Gapdh*
6.	F_1	5 month-old	Males	Hippocampus	*Gapdh*
7.	F_1	5 month-old	Females	Cortex	*Gapdh*
8.	F_1	5 month-old	Females	Hippocampus	*Gapdh*
9.	F_2	1 month-old	Males	Cortex	*Actb*
10.	F_2	1 month-old	Males	Hippocampus	*Gapdh*
11.	F_2	1 month-old	Females	Cortex	*Gapdh*
12.	F_2	1 month-old	Females	Hippocampus	*Gapdh*

1. Cortex of F_1 3 month-old males

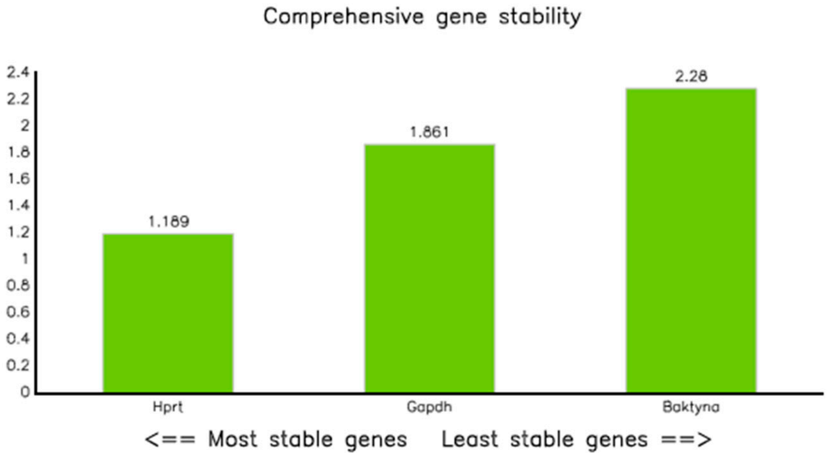

2. Hippocampus of F_1 3 month-old males

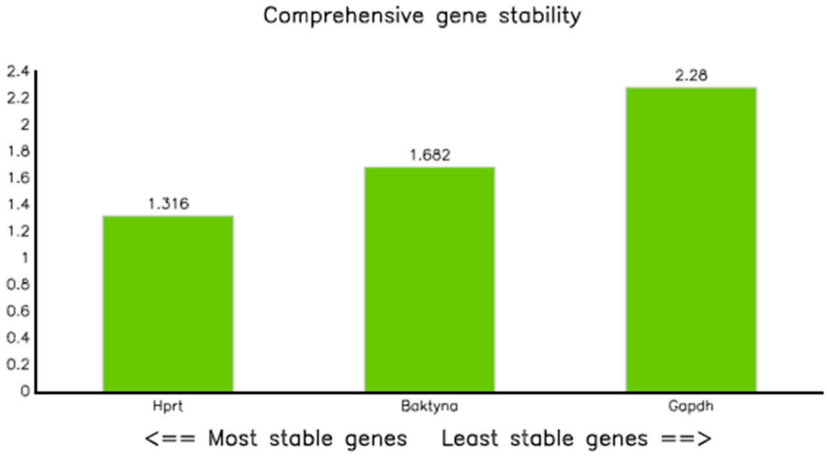

3. Cortex of F_1 3 month-old females

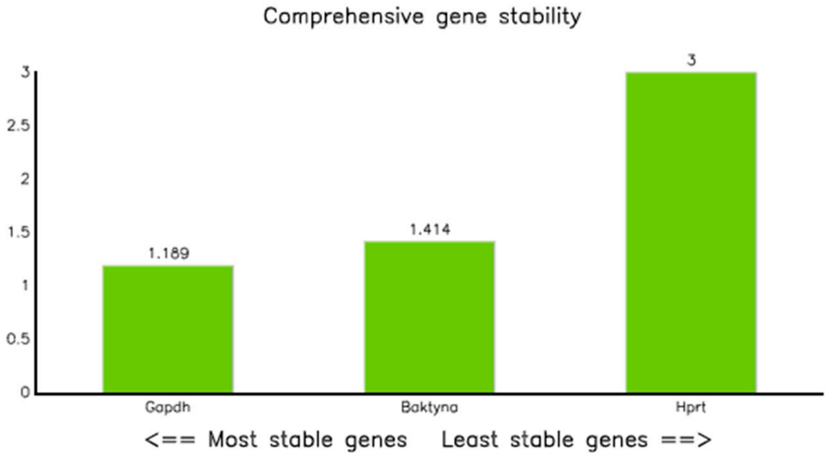

4. Hippocampus of F_1 3 month-old females

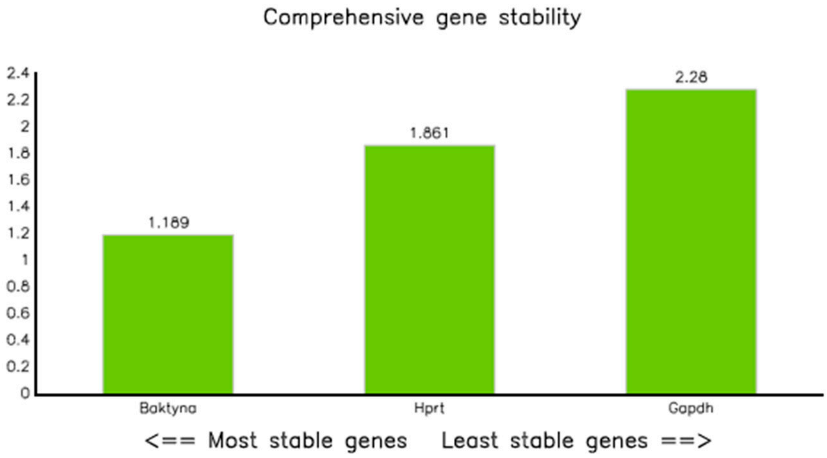

5. Cortex of F_1 5 month-old males

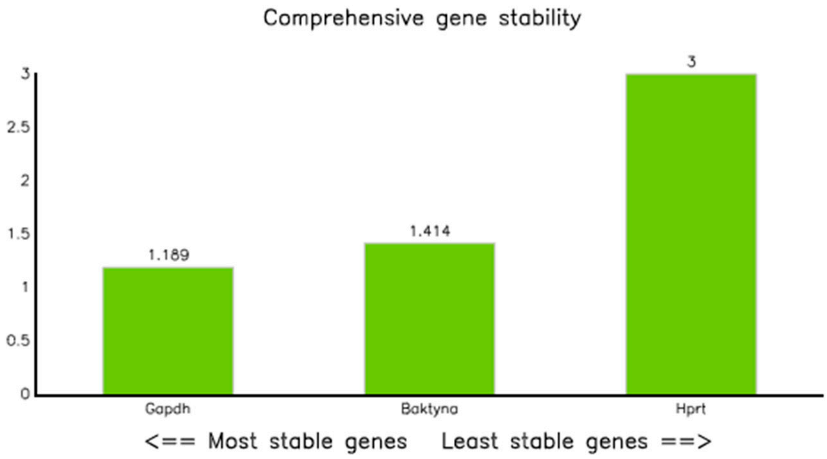

6. Hippocampus of F_1 5 month-old males

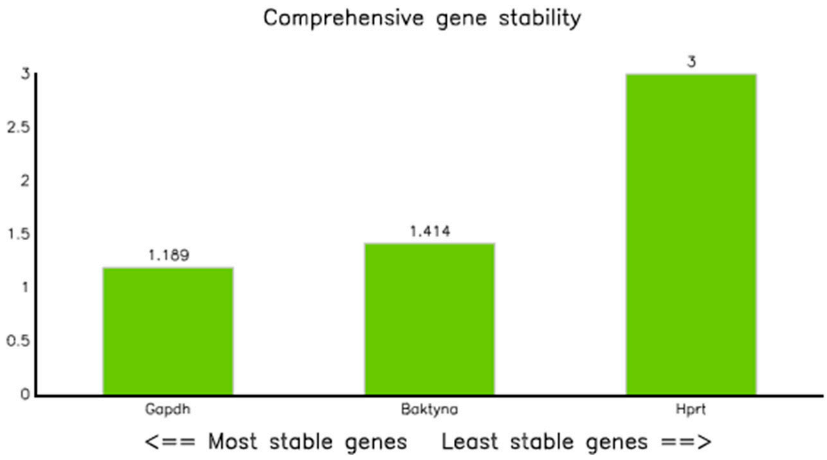

7. Cortex of F_1 5 month-old females

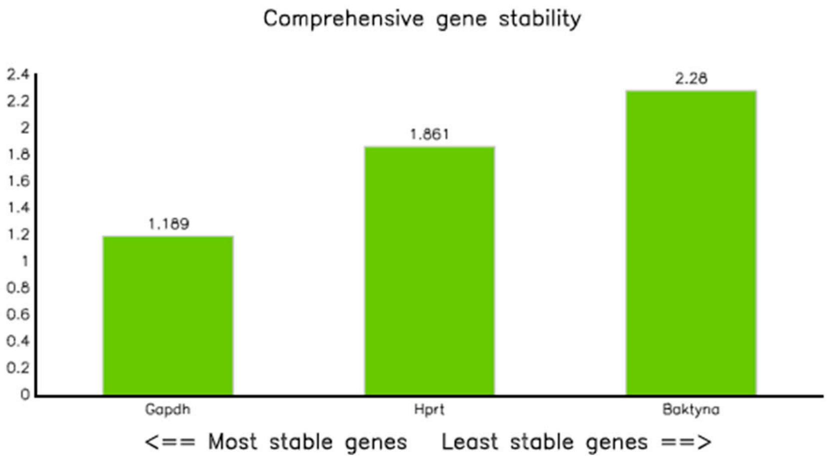

8. Hippocampus of F_1 5 month-old females

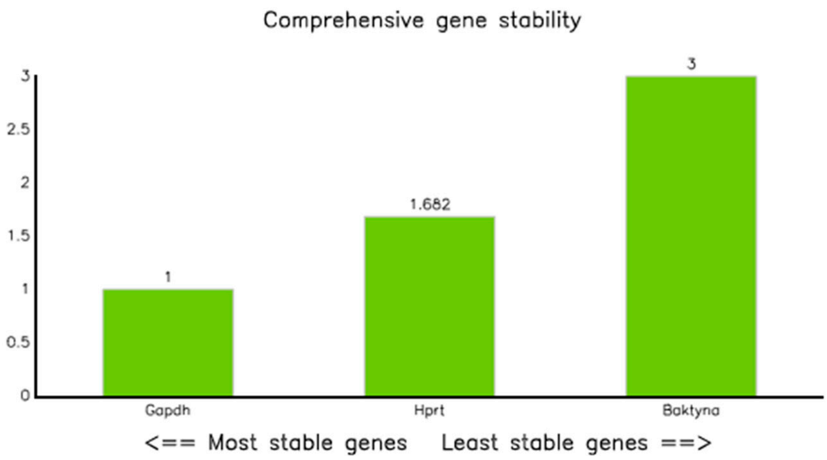

9. Cortex of F_2 1 month-old males

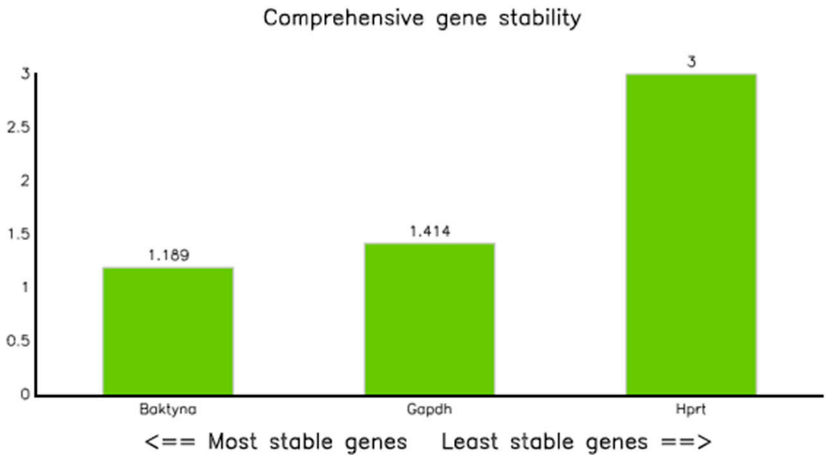

10. Hippocampus of F_2 1 month-old males

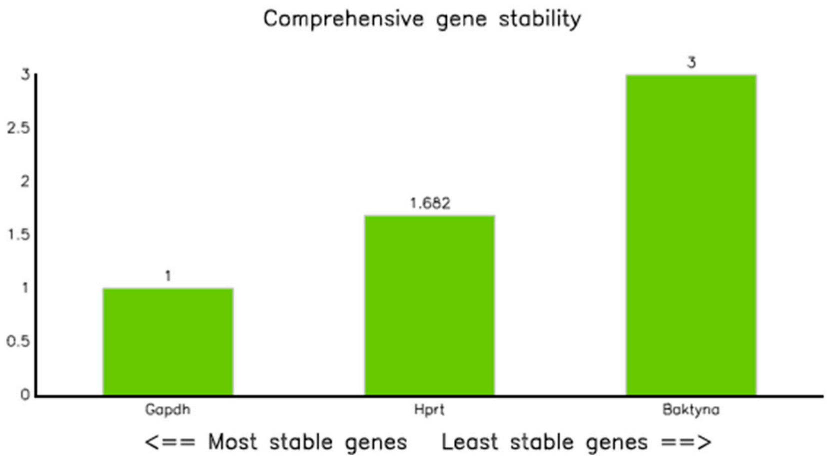

11. Cortex of F_2 1 month-old females

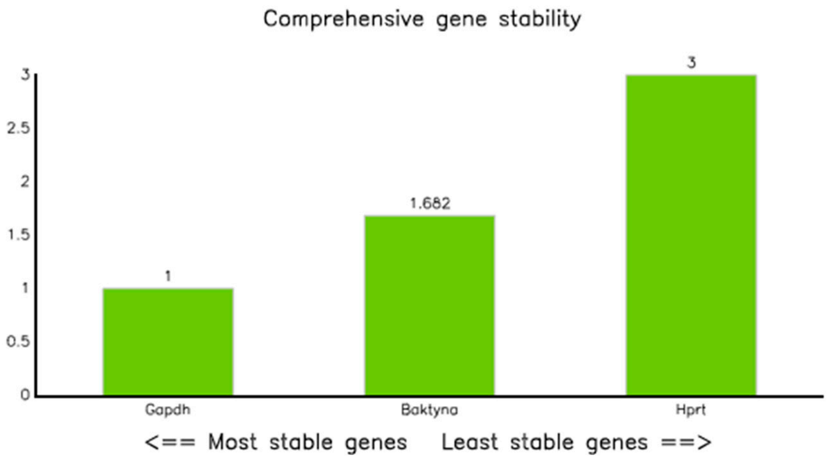

12. Hippocampus of F_2 1 month-old females

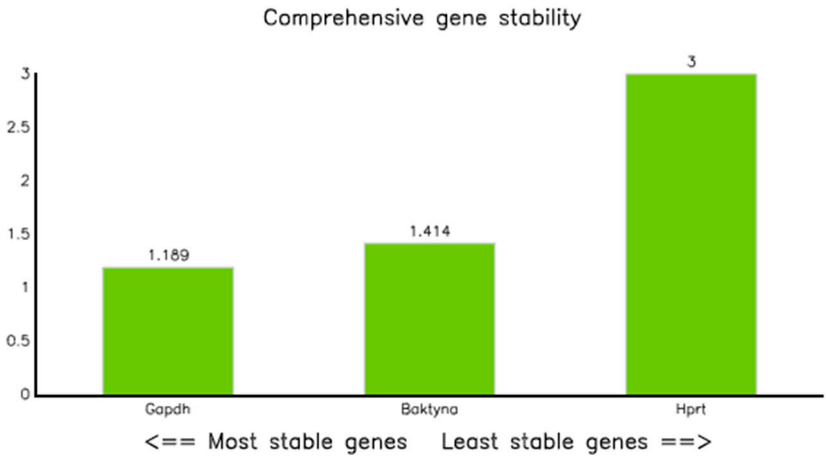

Supplement: Supplementary file 1 [file toxics-12-00906-s001.zip › toxics-3328472-supplementary.pdf]
